# Emerging Links between Control of Mitochondrial Protein ATAD3A and Cancer

**DOI:** 10.3390/ijms21217917

**Published:** 2020-10-25

**Authors:** Liwei Lang, Reid Loveless, Yong Teng

**Affiliations:** 1 Department of Oral Biology and Diagnostic Sciences, Dental College of Georgia, Augusta University, Augusta, GA 30912, USA; llang@augusta.edu (L.L.); RLOVELESS@augusta.edu (R.L.); 2 Georgia Cancer Center, Department of Biochemistry and Molecular Biology, Medical College of Georgia, Augusta University, Augusta, GA 30912, USA; 3 Department of Medical Laboratory, Imaging and Radiologic Sciences, College of Allied Health, Augusta University, Augusta, GA 30912, USA

**Keywords:** ATAD3A, mitochondria, cancer, metabolism, drug target

## Abstract

Spanning from the mitochondria’s outer surface to the inner membrane, the nuclear-encoded protein ATAD3A maintains vital roles in regulating mitochondrial dynamics, homeostasis, metabolism, and interactions with the endoplasmic reticulum. Recently, elevated levels of ATAD3A have been reported in several types of cancer and to be tightly correlated with cancer development and progression, including increased cancer cell potential of proliferation, metastasis, and resistance to chemotherapy and radiotherapy. In the current review, we reveal ATAD3A as the link between mitochondrial functions and cancer biology and the accumulating evidence presenting ATAD3A as an attractive target for the development of novel cancer therapy to inhibit aberrant cancer metabolism and progression.

## 1. Introduction

In addition to their bioenergetic role, mitochondria function as signaling platforms and key regulators of cellular processes related to biosynthesis, Ca^2+^ homeostasis, and cell death. In cancer, mitochondrial function is critical to cell survival through genetic and/or environmental events, leading to metabolic reprogramming and changes in mitochondrial biogenesis, mitophagy, and dynamics. While the function of mitochondria in cancer has historically been restricted to Warburg’s hypothesis (aerobic glycolysis), work over recent decades has served to help dispel misconceptions and deepen our understanding of the diverse and dynamic roles that these organelles play throughout tumor progression, such as in cell survival, proliferation, stemness, motility, metastasis, and therapeutic resistance [1,2]. Non-specific targeting of mitochondrial functions in the treatment of cancer, however, may have major unwarranted effects, like inhibition of normal cell growth. Therefore, refined strategies that allow for the specific functional blocking of oncoproteins that physically localize to the mitochondria in cancer cells will have to be devised for therapeutic intervention. Yet, given that there is no simple canon for the role of mitochondrial oncoproteins in the regulation of malignant mitochondrial programs, gaining mechanistic insights into these proteins and their respective signaling networks involved in tumor development and progression will be critical to the clinical exploration of novel anticancer therapies.

Mitochondrial ATPase family AAA domain-containing protein 3 (ATAD3) belongs to AAA+ (ATPases associated with various cellular activities) superfamily, which shares a highly conserved module for ATP hydrolysis and participates in a variety of cellular processes [3,4]. ATAD3 only exists in eukaryotic organisms and has three family members: ATAD3A, ATAD3B, and ATAD3C. Among them, ATAD3B and ATAD3C only exist in primates and humans. While, ATAD3B may act as a dominant negative inhibitor to ATAD3A function [5], the exact role of ATAD3C remains unknown. ATAD3A is believed to be the ancestral form and to be duplicated twice to form ATAD3B and ATAD3C [6]. Structurally, the ATAD3A protein spans the mitochondrial outer membrane (OM) and inner membrane (IM) and regulates dynamic interactions between the two that is sensed by cell fission machinery [4]. As a mitochondrial protein with the capacity to impact essential mitochondrial functions and organization, ATAD3A controls a broad spectrum of physiological and pathological responses, including mitochondrial dynamics, nucleoid organization, signaling transduction, and cholesterol metabolism [7,8,9]. ATAD3A mutations can cause a range of different phenotypes and have been identified as one of the most common causes of lethal infantile mitochondrial disease [10]. Although there have been no or few ATAD3A mutations identified in cancer patients, ATAD3A has nevertheless been implicated in certain types of cancer, where its elevated expression levels have been associated with poor patient outcome. Indeed, the aberrant activation of ATAD3A in cancer cells drives mitochondrial oncogenic signaling, leading to enhanced tumor-promoting activities. In this review, our current understandings of ATAD3A in cancer development and progression have been outlined.

## 2. The Essential Role of ATAD3A in Mitochondria

### 2.1. The Molecular Structure of ATAD3A in Mitochondria

**Figure 1:**
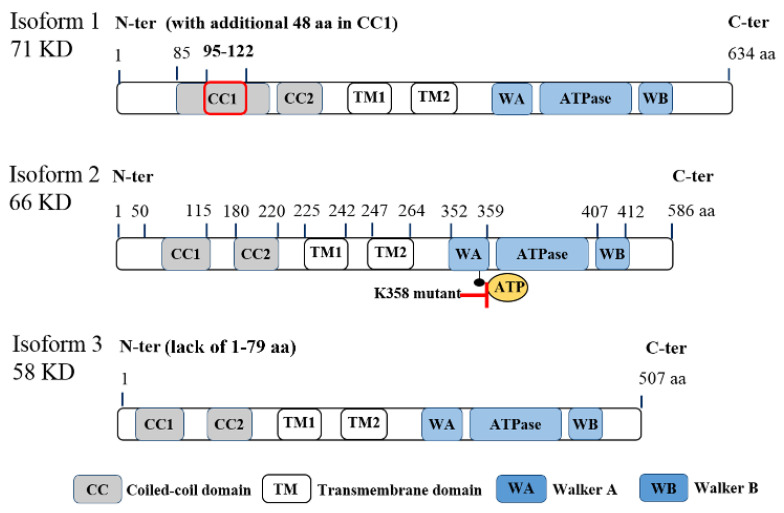
Molecular structure of ATPase family AAA domain-containing protein 3A (ATAD3A) transcript variants with vital domains. Schematic diagram for the molecular structure of three transcript variants of ATAD3A. Three isoforms have similar major domains, including two coiled-coil domains (CC1 and CC2), two transmembrane domains (TM1 and TM2), walker A (WA) and walker B (WB) domains for the ATP binding, and ATPase domain. Isoform 2 (66KD) is the major form in cancer cells. Compared with isoform 2, isoform 1 has an additional 48 amino acids in the CC1 domain, which may disrupt its function to form the oligomers or interaction with other mitochondrial partners. Isoform 3 lacks the first 50 amino acids in the N-terminal, which locates on the mitochondria surface and is essential for the interaction with cytoplasmic proteins. Of note, mutations on K355 or K358 in the WA domain markedly reduce the ATPase activity of ATAD3A.

ATAD3A is located on chromosome 1 at 1p36.33 locus and has three transcript variants, with isoform 2 being the major one with 586 amino acids (a.a.) (Figure 1). While the first 50 amino acids of the protein’s N-terminal can be found on the mitochondrial surface, there are several important domains within the N-terminal, including a flexible proline-rich region for possible protein–protein interactions (a.a. 18–27), transmembrane domain 1 (TM1, a.a. 225–242) for integrating the mitochondrial OM, transmembrane domain 2 (TM2, a.a. 247–264) for integrating the mitochondrial IM, and two coiled-coil regions (CC1, a.a. 85 to 115; CC2, a.a. 180–220) for the oligomerization of ATAD3A monomers and/or for interaction with other proteins [11]. Deacetylation on lysine 135 (K135) residue of ATAD3A is required for its oligomerization, especially for dimerization [12]. The ATPase domain and two ATP binding domains, Walker A (WA) and Walker B (WB), are located at the C-terminal of ATAD3A [5,13] (Figure 1). In particular, mutations on K355 or K358 of the WA domain can block the binding of ATPs to ATAD3A, subsequently influencing ATAD3A’s ATP affinity and reducing its ATPase activity [14]. Of note, these two mutations have been found to be disease-relevant and dominantly inherited in a family with hereditary spastic paraplegia [14]. However, high levels of ATAD3A expression, rather than ATAD3A mutations, have historically been identified in cancer patients [4]. Aside, it is worth mentioning that both the N-terminal and C-terminal regions of ATAD3A have been suggested to contribute to the protein’s interaction with S100B, a zinc and calcium-binding protein with a chaperone-associated function contributing to proper ATAD3A protein folding [15]. Interestingly, while the role of S100B in cancer is not yet well understood, interrogation of its interactions with target proteins like p53 [16] and the potential to serve as a marker for metastasis in different cancers has been reported [17].

### 2.2. Functions of ATAD3A in Mitochondria Homeostasis

**Figure 2:**
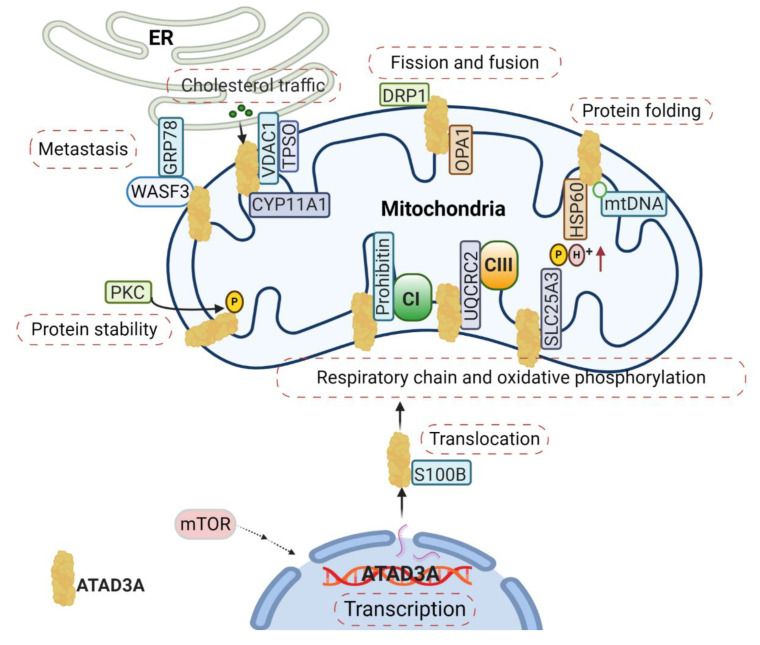
The complexity of the ATAD3A signaling network in mitochondria. The main signaling pathways involve (1) the regulation of ATAD3A expression levels by mTOR, (2) the proper folding and mitochondrial localization of newly synthesized ATAD3A protein associated with S100B’s function, (3) ATAD3A protein stability regulation by PKC, (4) and communication between the endoplasmic reticulum (ER) and mitochondria mediated by the ATAD3A/WASF3/GPR78 axis. Moreover, ATAD3A has been identified as one essential part of transduceosome, also known as the cholesterol transfer complex. ATAD3A has the ability to assist with the transportation and metabolism of cholesterol by interacting with voltage-dependent anion channel (VDAC) and CYP11A1 in mitochondria. ATAD3A governs mitochondrial dynamics through the functional regulations of mitochondrial fission- dynamin-related protein 1 (DRP1) and fusion protein-OPA1. ATAD3A also contributes to mitochondrial respiration via interactions with several important respiration proteins, such as prohibitin, UQCRC2 and SLC25A3.

Mitochondria function is tightly associated with its dynamics, including mitochondrial fission and fusion. ATAD3A regulates mitochondrial dynamics through its interactions with mitochondrial fission (dynamin-related protein 1, DRP1) and fusion (mitofusins, OPA1) proteins [18] (Figure 2). Silencing *ATAD3A* by small interfering RNAs (siRNAs) or ectopic expression of deficient mutant ATAD3A increases mitochondrial fragmentation, while knockdown of *DRP1* eliminates mitochondrial fragmentation [14,19]. In addition to its involvement with mitochondrial fusion and shaping, mitofusin-2 is critical for maintaining close mitochondrial interaction with the endoplasmic reticulum (ER) [20]; when mitofusion-2 is depleted, ATAD3A localization to mitochondrial-associated membranes is increased [21]. As an ATP-dependent chaperonin, HSP60 mediates mitochondrial proteostasis with its co-chaperonin HSP10. In particular, the interaction of ATAD3A and HSP60 has been detected at ATAD3A’s C-terminal [8] (Figure 2). ATP binding-deficient ATAD3A harbors a mutation in its WA domain, leading to mitochondrial fragmentation in glioblastoma cells [8].

Mitochondrial DNA (mtDNA) is organized in the nucleoprotein complex associated with the IMM. mtDNA segregation and changes in mitochondrial architecture can be induced by altering the structure or composition of the nucleoid [22]. Interestingly, altered ATAD3A expression perturbs mtDNA maintenance and replication [23]. A more recent study shows that ATAD3A is a detergent-resistant component that organizes mtDNA and segregates mitochondrial nucleoids, and that ATAD3A deficiency leads to modifications in mtDNA organization [24]. Moreover, loss of ATAD3A induces early and severe mitochondrial structural abnormalities, progressive mtDNA depletion and deletions, and muscle atrophy in mice [25]. Loss of ATAD3A also leads to a dramatic reduction in mitochondrial cristae junctions and changes in cristae morphology [25]. Further, ATAD3A plays a critical role in regulating IMM structure, leading to secondary defects in mtDNA replication, complex V, and cholesterol levels [25]. Interestingly, in later work evaluating human siblings with a recessive missense ATAD3A mutation that likely disrupts WB, it was found that while the patient’s fibroblast possessed mitochondrial cristae malformations alongside decreased ATAD3A levels, in contrast to knockout mouse models, no changes in oxidative phosphorylation complexes were seen [26]. Importantly, ATAD3A has been reported to maintain homeostasis in mouse hematopoietic cells by impeding Pink1 mitophagy. Here, it was seen that the deletion of ATAD3A hyperactivates mitophagy by facilitating Pink1 transportation and activity [27].

Mitochondria–ER interactions are critical to enabling mitochondrial adaptation and maintaining organelle homeostasis. Particularly, the ER supplies biomolecules needed for biogenesis and helps govern numerous processes, such as those involved in stress and morphological changes [28]. Given the critical link between these two organelles, it has been suggested that ATAD3, as a contact site, could represent an evolutionary step towards mitochondrial adaptation to ER interactions [29]. Nevertheless, ATAD3A’s various involvements and precise functions have yet to be fully elucidated.

### 2.3. The Role of ATAD3A in Mitochondrial Metabolism and Respiration

From Nematoda to mammalian, ATAD3A is critical in the development of a number of multicellular organisms. Silencing *ATAD3A* in *C. elegans* and *Drosophila* induces growth arrest in larvae [7,8]. In murine embryos, knockout of *ATAD3A* is lethal, causing retardation and defects in trophoblast lineages, possibly due to low mitochondrial biogenesis and ATP production [9]. Deletion or mutation of ATAD3A in the WA domain has also been linked to distinct neurological syndromes in humans, including global developmental delay, hypotonia, optic atrophy, axonal neuropathy, and hypertrophic cardiomyopathy [14,30]. Steroid hormones are synthesized in the mitochondria and smooth ER by a variety of tissues, such as the adrenal cortex, gonads, and placenta. These hormones are all derived from cholesterol and influence the development and progression of human cancers by binding steroid hormone receptors (SHRs) [31,32]. Cholesterol trafficking occurs between the ER and mitochondria, where communication between the two organelles facilitates both steroidogenesis substrate availability and mitochondria product passage to different steroidogenic enzymes in the ER [33]. During steroidogenesis, the rate-limiting step is the transfer of cholesterol from the OMM to the IMM, where it is converted into pregnenolone by the cytochrome P450 enzyme CYP11A1 [34] (Figure 2). Alongside the voltage-dependent anion channel (VDAC) and other constituents like cytosolic proteins, ATAD3A has been identified as an essential component of the transduceosome complex through which this transport of cholesterol is facilitated [34] (Figure 2).

In MA-10 mouse tumor Leydig cells, knockdown of *ATAD3A* leads to a significant decrease in steroid production [33]; and in patients with *ATAD3* gene cluster deletions, derived fibroblasts display abnormalities in cholesterol metabolism [24]. Our studies show that the ATAD3A-WASF3-GRP78 axis, which bridges the interaction between the mitochondria and ER, may possess a potential role in the regulation of cholesterol traffic [35]. In addition, the critical role of ATAD3A in mitochondria metabolism, especially of lipids, has been confirmed in several kinds of model organisms. In *C. elegans*, reduction of intestinal fat storage and low lysosomal content have been reported when *ATAD3A* is knocked down [7]. Interestingly, it has been revealed that both the N-terminal and C-terminal of ATAD3A are required for normal cell growth and cholesterol channeling in *Drosophila* [8]. Lastly, altered cholesterol metabolism was reported in the skeletal muscle of conditional *ATAD3A* knockout mice [25].

It has also been demonstrated that ATAD3A participates in mitochondrial respiration [27]. In *C. elegans*, silencing *ATAD3A* decreases levels of both complex I and citrate synthase, diminishing mitochondrial activity and ultimately impeding larval development [7]. In mouse hematopoietic cells, knockout of *ATAD3A* results in decreased mitochondrial mass and impaired mitochondrial functions, with abnormalities seen through lower rates of basal oxygen-consumption and diminished oxidative capacity [27]. Several interactions between ATAD3A and components critical to mitochondrial respiration have been identified and include prohibitin, UQCRC2, and SLC25A3 [21] (Figure 2). Prohibitin associates and stabilizes respiratory complexes, particularly Complex I, and regulates the proteolysis of unassembled IMM proteins of the oxidative phosphorylation system [36]. As a core subunit of Complex III, UQCRC2 is needed for complex III’s conversion into its catalytically active homodimer form, which can subsequently be incorporated into a larger supercomplex that functions as one enzyme [37]. SLC25A3 is located in the IMM and serves to transports phosphate groups (along with H+) from the cytosol to the mitochondrial matrix during oxidative phosphorylation [38]. The ways by which ATAD3A regulates mitochondrial respiration through these protein interactions, however, remains unclear. Notably, numerous other respiratory complex components have been found to be directly or indirectly related to ATAD3A, further obscuring the protein’s exact roles. Particularly, *ATAD3A* knockdown in mouse 3T3-L is seen to decrease the expression, for example, of MTCO1, MTCO2, ATP5A, UQCRC2, SDHB, NDUFB8, and NDUFA10 [33].

## 3. ATAD3A in Cancer

### 3.1. Elevated ATAD3A Expression in Cancers

Elevated levels of ATAD3A have been found in lung adenocarcinomas, prostate cancer, head and neck cancer, gliomas, uterine cervical cancer, and breast cancer [18,35,39,40,41,42]. Upregulation of transcription and increased protein stability (post-translation modification) are potential mechanisms for elevated ATAD3A in cancer, as TCGA data analysis indicates that *ATAD3A* gene amplification and mutation is rare in cancer cells [4]. The promoter region of the *ATAD3A* gene contains several regulatory elements for cell growth, such as C/EBP, CBF/IRP/CREB, GATA-1, Oct-1, and TFIID [5]. Still, the regulation of ATAD3A in the context of the tumor microenvironment remains poorly understood. In particular, limited overall blood flow is typical in solid tumors due to highly abnormal tumor vasculature [43]. Upregulated ATAD3A expression can be induced by serum starvation in lung cancer cells, which is related to the gene’s translation rather than its transcription [18]. Another potential mechanism for increased ATAD3A levels in cancer is an increase in protein stability, which may be regulated by protein kinases [18]. According to the analysis by PhosphoSitePlus^®^, 10 putative phosphorylation sites on ATAD3A have the potential to be modified by protein kinases, including PKC, PKA, GSK3, cdc2, CKI, CKII, DNAPK, RSK, Cdk5, PKG, p38MAPK, and INSR [4,5]. In one study, ATAD3A phosphorylation by PKC, a prototypical class of serine/threonine kinases participating in cancer progression [44], was confirmed. Specifically, Thr335, Thr338, and Thr359 have been identified as the three putative phosphorylation sites for PKC on ATAD3A isoform 2 (Figure 2). Moreover, ATAD3A expression can be upregulated by PKC. Employment of the PKC inhibitor Calphostin C decreases ATAD3A expression, whereas ectopic expression of PKC isozymes increases the expression of ATAD3A in A549 and H23 lung cancer cells [18]. Analysis through the online tool Ubinet^®^ suggests that K358 and K573 are the two putative binding sites for ubiquitin on ATAD3A (isoform 2) [45]; however, the underlying mechanisms of ATAD3A expression in cancer remain to be explored.

### 3.2. ATAD3A Promotes Tumor Growth

Increasing evidence has indicated ATAD3A to be essential for cancer cell proliferation and tumor growth. Previous work from our group has demonstrated that silencing *ATAD3A* by shRNA inhibits cell anchorage-independent growth of MDA-MB-231 breast cancer cells and SW620 colon cancer cells. Moreover, knockdown of *ATAD3A* suppresses breast tumor growth in vivo [35]. Overexpression of ATAD3A is also associated with increased cell proliferation in glioma [41]. Nevertheless, the potential signaling pathways regulated by ATAD3A in cancer cell proliferation remain poorly understood. Of note, target of rapamycin (TOR), a serine/threonine-protein kinase known to regulate nuclear mitochondrial protein expression and to play an essential role in cell proliferation control [11], has been found to modulate mitochondrial functions through ATAD3A in several model organisms in *drosophila*, for example, drosophila ATAD3A (dATAD3A) is regulated by the TOR signaling pathway and is involved in cell growth and division [46]. Either genetic depletion of *dATAD3A* or suppression of its expression by mtor inhibitor rapamycin induces larval growth arrest and reduces the size of fat body cells [8]. Moreover, the ATAD3A/TOR axis is critical for mitochondrial biogenesis during mice embryo development. Loss of *ATAD3A* or mTOR leads to defective embryo development and early death [9]. Notably, *ATAD3A* has been found to positively upregulate the expression of mTOR, sterol regulatory element-binding protein (SREBP-1c), and Cyclin D1 in dairy cows, which are involved in milk biosynthesis and cell proliferation [47]. Certain tumor suppressors may also regulate cancer cell growth through interaction with ATAD3A. Recently, ATAD3A was identified to be involved in fat 1 protein (FAT1)-interaction by tandem affinity purification and mass spectrometry (TAP-MS ). FAT1 is a well-studied tumor suppressor that plays a vital role in cell growth control and mitochondria function [48]. Smooth muscle cells tend to grow faster and consume more oxygen for atp production when deficient in FAT1 [48]. It is possible that the functional interaction between FAT1 and ATAD3A is one of the critical molecular mechanisms underpinning ATAD3A-induced tumor growth.

### 3.3. ATAD3A Facilitates Cancer Metastasis

A positive correlation between ATAD3A and cancer metastasis has been identified in several types of cancer, such as prostate cancer, urothelial carcinoma (UCC), lung adenocarcinoma (LADC), and breast cancer [18,35,39,42]. In the case of prostate cancer, increased ATAD3A correlates with tumor grade, disease status, lymphovascular infiltration, serum PSA levels, as well as expression of the androgen receptor (AR) [39]. The expression of ATAD3A also has a correlation among the presence of hrHPV, disease stage, lymph node involvement, and patient survival in patients with UCC [42]. Similarly, higher levels of ATAD3A are associated with reduced overall survival in patients with hepatocellular carcinoma [49] and lower survival time in patients with LADC [18]. In addition to correlating with tumor stage and lymphovascular infiltration, high ATAD3A expression in LADC patients is correlated with relapse rates three-fold higher than those with low ATAD3A expression [18]. Although accumulating evidence indicates that ATAD3A plays a vital role in cancer metastasis, the underlying mechanisms of dissemination related to ATAD3A remain largely unknown. Our recent study demonstrates that ATAD3A regulates the invasion and metastatic potential of breast cancer cells through interacting with Wiskott–Aldridge syndrome family protein 3 (WASF3) [35]. In particular, overexpression of ATAD3A advances breast cancer metastasis by increasing the stability of WASF3 protein, a well-studied tumor metastasis promoter [35]. WASF3 regulates cancer cell movement and invasion through suppression of the metastasis suppressor KISS1 [35] among other mechanisms, and eventually facilitates actin polymerization through the recruitment of ARP2/3 complexes [50,51]. ATAD3A is a novel interacting partner of WASF3 and acts as a crucial mediator to promote cell invasion in breast cancer by regulating the stabilization of WASF3 with the ER protein GPR78, a resident chaperone involved in protein degradation (Figure 2) [52]. *ATAD3A* silencing reduces GRP78 expression and, thus, disrupts the interaction between WASF3 and GRP78, ultimately initiating WASF3 protein degradation. The gene expression profiling from microarray analysis showed that the expression levels of *E-Cadherin/CDH1*, a critical gene in maintaining cell–cell adhesion and epithelial–mesenchymal transition [53], were significantly upregulated in breast cancer cells when *ATAD3A* was knocked down. It appears that E-Cadherin is one of the downstream targets of WASF3 in breast cancer cells [54] and impaired invasion of *ATAD3A* knockdown cells can be rescued by restoring WASF3 protein function. These novel findings strongly suggest that ATAD3A may exert its effect on E-Cadherin through a WASF3-dependent mechanism.

## 4. ATAD3A in Other Diseases

Given ATAD3A’s essential roles in mitochondrial maintenance and biogenesis, it is unsurprising that *ATAD3A* gene mutations, deletions, and structural variants have been identified and linked to human disease. Notably, both dominant and recessive mechanisms of *ATAD3* gene alteration exists and give rise to distinct phenotypes of various intensities. Recently, variants at the *ATAD3* gene locus have been linked to Harel–Yoon syndrome, a neurodevelopmental disordered characterized by delayed psychomotor development, truncal hypotonia, and peripheral neuropathy, among other clinical features. In particular, an identical de novo heterozygous ATAD3A c.1582C>T (p.Arg528Trp) mutation was reported in five unrelated individuals that presented with these phenotypes [30]. In *drosophila*, it was found that overexpression of this mutation causes a significant decrease in mitochondrial content, abnormal mitochondrial morphology, and increased autophagy. In two additional families, biallelic variants in ATAD3A were also identified. In the first family, two siblings with ataxia, seizures, and hypotonia were found to possess a homozygous ATAD3 single-nucleotide variant (Thr53Ile). In the second family, the subject, who died at 13 days, was found to have recessive copy-number variant deletion of *ATAD3A* (exons 1–5) mediated by nonallelic homologous recombination (NAHR) between ATAD3 paralogs [30].

Using whole-exome sequencing, another dominantly inherited heterozygous variant c.1064G > A (p.Gly355Asp) in ATAD3A was identified in a mother with hereditary spastic paraplegia and axonal neuropathy [14]. In particular, this dominant-negative mutation affects WA, leading to reduced ATPase activity and mitochondrial network fragmentation when overexpressed. Moreover, this mutation was associated with increased lysosomes in patient neurons and fibroblasts derived through differentiation of patient-specific induced pluripotent cells [14]. More recently, the homozygous variant c.1217T>G (p.Leu406Arg) in ATAD3A was identified in four siblings presenting with fatal neonatal cerebellar hypoplasia, seizures, axial hypotonia, hypertrophic, cardiomyopathy, hepatomegaly, congenital cataract, and dysmorphic facies [26]. In consequence of this missense mutation, it is predicted that a polar side is introduced into the catalytic domain of ATAD3A and influences protein stability. In effect, fibroblasts derived from these individuals were seen to have decreased levels of ATAD3A with significant ultrastructural alterations of mitochondrial cristae and morphology, underscoring the essential role of ATAD3A in mitochondrial biogenesis [26]. An additional report of Harel–Yoon syndrome has also been found in a 3-month-old with the *ATAD3* gene variant c. 1726C>T (p. Arg576Trp) [55]. Furthermore, the recessively inherited ATAD3 variants c.1609 T>A (p.Trp537Arg) and c.1614 + 2_1616 + 16del (p.Arg503Profs*11) have been identified in two siblings [56].

Rearrangements in the *ATAD3A-C* gene cluster region have also been of great interest. Specifically, in four unrelated individuals, it was found that biallelic deletions at the *ATAD3* locus generated chimeric *ATAD3A/ATAD3B* fusion genes that caused congenital pontocerebellar hypoplasia [24]. On the other hand, when *ATAD3* gene rearrangements affected the *ATAD3B*/*ATAD3C* genes on one allele and the *ATAD3A*/*ATAD3B* genes on the other, late-onset encephalopathy with cerebellar atrophy, ataxia, and dystonia was seen as the clinical effect. Notably, fibroblasts from these affected individuals indicated abnormalities in mitochondrial DNA and cholesterol metabolism [24]. In five neonates, de novo monoallelic reciprocal duplication at the ATAD3 locus, likely mediated by non-allelic homologous recombination, has been reported [57]. These individuals possess a lethal metabolic disorder characterized by cardiomyopathy, corneal opacities, encephalopathy, hypotonia, and seizures. In particular, this duplication produces an *ATAD3A/ATAD3C* fusion gene product that is functionally impaired and similarly leads to abnormalities in mitochondrial DNA organization and cholesterol metabolism. Importantly, results from this study provided an additional mutational mechanism for *ATAD3* gene cluster variants and highlighted the importance of copy number analysis [57]. Of note, six different de novo duplications in the ATAD3 locus have further been reported in 17 patients from 16 families [10]. Here, an extra copy of the *ATAD3B* gene and an *ATAD3A*/*ATAD3C* fusion gene, whose stable protein product interrupts ATAD3 oligomerization, are formed. These duplicates are associated with lethal perinatal cardiomyopathy, persistent hyperlactacidemia, corneal clouding or cataracts, and encephalopathy. Specifically, a decrease in oxidation phosphorylation complex I and its activity in heart tissue were observed [10].

In one child, a homozygous variant in the *ATAD3A* splice region has been found to correspond to a loss of function variant [58]. In this case, the patient was reported to experience normal neurodevelopment for the first 5 months of her life, then presented with axial hypotonia, hyporeflexia, and weakness, with clear developmental regression at 8 months, ophthalmoplegia at 18 months, epilepsy partialis continua at 21 months, and central and obstructive apneas alongside cerebral atrophy by 24 months. For this patient, it is predicted that a “leaky” splice variant allowed for some degree of ATAD3A function, causing a slower clinical progression [58]. Although the exact function and consequences of ATAD3A disruption remain unknown, technologies like whole-exome, whole-genome, and long-read DNA sequencing have begun to provide us insight into its genetic mechanisms and gain a more accurate understanding of its inheritance.

## 5. ATAD3A and Treatment Responses

### 5.1. ATAD3A Is Engaged in Chemo-Resistance

The *ATAD3A* gene is potentially associated with cancer response to chemotherapy. For example, high expression levels of ATAD3A have been observed in LDAC, and silencing it in these cells leads to increased sensitivity to the chemo agent cisplatin [18]. HPV E6 and E7 oncoproteins are necessary for malignant conversion in UCC [59]. Interestingly, E6 and E7 viral proteins upregulate ATAD3A expression which hinders cell autophagy and apoptosis and leads to chemodrug resistance in UCC patients [42]. Increased expression levels of ATAD3A also leads to the resistance of glioma cells to doxorubicin and temozolomide, and inhibiting it increases chemosensitivity in this cancer type [60]. Moreover, resistance to cisplatin treatment is decreased in LNCaP cells when *ATAD3A* is silenced [39].

### 5.2. ATAD3A Is Engaged in Radio-Resistance

ATAD3A is also involved in cancer resistance to radiotherapy. One study shows that ectopic overexpression of *ATAD3A* increases the resistance of glioblastoma multiforme to irradiation [60]. Upon *ATAD3A* silencing, however, these cells became more sensitive to radiation and were observed to lack double-stranded break repairs, manipulating ATM and H2AX compartmentalization. Moreover, DNA repair-related genes, including *H2AFX*, *Rad9B*, *Hus1B*, *MSH4*, and *LIG4*, were affected by the silencing of *ATAD3A* [60]. In prostate cancer, ATAD3A has also been shown to behave as an anti-apoptotic factor that promotes cancer cell survival upon radiotherapy [39].

## 6. Anti-Cancer Targeting of ATAD3A and Family Members

### 6.1. ATAD3A as a Potential Novel Target for Cancer Therapy

Considering that the role of ATAD3A in cancer development, progression and treatment outcomes has been experimentally proven, its targeting has emerged as a promising anti-cancer strategy. Although Calphostin C and resveratrol have been reported to inhibit ATAD3A expression in lung cancer cells [18,61], these approaches do not directly inhibit ATAD3A; in fact, no specific inhibitors targeting ATAD3A are currently available. Therefore, there is significant room for the development of novel ATAD3A targeting approaches, such as through the use of proteolysis targeting chimera (PROTAC) to decrease ATAD3A stability via selective intracellular proteolysis, cyclic peptides to interrupt ATAD3A–protein interactions, and antisense oligonucleotides or miRNA approaches to target *ATAD3A* gene expression. In particular, because the intact portions of the ATPase domain and binding regions are required for ATAD3A function in cancer cells, several approaches have been developed around the inhibition of these portion’s transcription and translation to block ATAD3A’s ATPase activity. While targeting the WA and WB domains of ATAD3A to disrupt its function is one therapeutic option, interrupting the critical bindings between ATAD3A and its interacting partners is another. DA1, a peptide that competitively binds to DRP1, can selectively disrupt ATAD3A–DRP1 interaction and eventually suppress mitochondrial fragmentation and mtDNA lesion; notably, this peptide has been used in Huntington’s disease mouse- and patient-derived cells to reduce bioenergetic deficits and cell death [12]. Further, the potential of DA1 in cancer treatment is currently being determined in our lab using preclinical animal models. To enable screening of novel inhibitors targeting ATAD3A transcription from a large number of drug-like bioactive compounds, we have also developed an ATAD3A-based in situ coincidence reporter [62]. In this approach, the expression of firefly luciferase (FLuc) and nanoluciferase (NLuc) are simultaneously driven by ATAD3A core promoter; and insertion of coincidence reporter genes in the target gene locus through homology-directed repair (HDR) can be efficiently enriched by puromycin selection [62]. This novel molecular reporter endows us to identify novel ATAD3A transcript inhibitors with high confidence, which should interest a range of cancer scientists and clinicians who seek to assess the feasibility of manipulating currently undruggable targets for therapeutic interventions.

### 6.2. ATAD3 Family Members and Potential Synergistic Anti-Cancer Targeting

Within the ATAD3 family, ATAD3B and ATAD3C present significant molecular and expressive differences when compared with ATAD3A. ATAD3B’s C-terminal, for example, is 62 amino acids longer than ATAD3A’s, and ATAD3C’s C-terminal is truncated. In humans, ATAD3A is also expressed ubiquitously while ATAD3B expression is typically only seen in embryos and specific parts of the brain. ATAD3B has been reported to be incorporated into ATAD3A oligomers and regulates the interaction of ATAD3A with matrix nucleoid complexes [5], but ATAD3C expression and interaction with ATAD3A and/or ATAD3B remain largely unknown. Most importantly, ATAD3B is an embryonic pluripotent stem cell-specific protein, which can be expressed to different extents in both small-cell carcinoma lines (e.g., H810) and the non-small-cell lung carcinoma lines (e.g., H358, H1975, and H1299) [5]. Overexpression of ATAD3B has also been found to be associated with lower survival of post-menopausal breast cancer patients with estrogen receptor α (ERα)^+^ tumors [63]. Considering that ATAD3B is a potential stem cell-specific marker expressed in certain cancer types, it is suggestive, therefore, that ATAD3B may be a putative target to complement anti-cancer therapeutics [11]. Mounting evidence suggests that many tumors include both tumor-amplifying cancer cells (which contribute to tumor mass) and cancer stem cells (with the potential to initiate tumorigenesis) may possibly serve in processes related to treatment resistance, metastasis, and recurrence. In this case, anti-cancer therapies co-targeting ATAD3A and ATAD3B would likely be required, though may give rise to an encouraging synergistic effect leading to greater therapeutic efficacy.

## 7. Conclusions

As a new mitochondrial AAA+ family member, ATAD3A is essential for mitochondrial homeostasis, lipid metabolism, and communication with the ER. Moreover, it is becoming increasingly clear that ATAD3A maintains grave importance to cancer development, progression, and resistance to chemo and radiotherapy. Nevertheless, the multiple roles of ATAD3A reflect the complete precision required to recognize the protein’s various regulators and effector molecules. Although there are many molecules and pathways (including PKC, WASF3, and FAT1) that regulate ATAD3A expression and function at different levels, obtaining a more complete description of these signaling networks will ultimately bolster our understanding of ATAD3A regulation. Another important challenge for the future is to understand how interactions between ATAD3A with other mitochondrial proteins and effectors are spatially and temporally regulated. Delineating both the biological and pathological roles of ATAD3A will then allow us to shed lights on the steps required to develop more effective anti-ATAD3A approaches for cancer treatment.

## Abbreviations

ATAD3A	ATPase family AAA domain-containing protein 3A
a.a.	amino acids
DRP1	dynamin-related protein 1
dATAD3A	drosophila ATAD3A
ER	endoplasmic reticulum
HNSCC	head and neck squamous carcinoma
FAT1	Fat 1 protein
HDR	homology-directed repair
IM	inner membrane
LADC	lung adenocarcinoma
mtDNA	mitochondrial DNA
SHRs	steroid hormone receptors
siRNA	small interfering RNA
OM	outer membrane
TAP-MS	tandem affinity purification and mass spectrometry
TM	transmembrane
TOR	target of rapamycin
UCC	urothelial carcinoma
VDAC	voltage-dependent anion channel
WA	Walker A
WASF3	Wiskott–Aldridge syndrome family protein 3
WB	Walker B
